# MHC Class I Stability is Modulated by Cell Surface Sialylation in Human Dendritic Cells

**DOI:** 10.3390/pharmaceutics12030249

**Published:** 2020-03-10

**Authors:** Zélia Silva, Tiago Ferro, Danielle Almeida, Helena Soares, José Alexandre Ferreira, Fanny M. Deschepper, Paul J. Hensbergen, Martina Pirro, Sandra J. van Vliet, Sebastian Springer, Paula A. Videira

**Affiliations:** 1 UCIBIO, Departamento Ciências da Vida, Faculdade de Ciências e Tecnologia, Universidade Nova de Lisboa, 2829-516 Caparica, Portugal; zm.silva@fct.unl.pt (Z.S.); tj.ferro@fct.unl.pt (T.F.); dsi.almeida@campus.fct.unl.pt (D.A.); f.deschepper@campus.fct.unl.pt (F.M.D.); 2 CDG & Allies – PPAIN- Congenital Disorders of Glycosylation & Allies - Professionals and Patient Associations International Network, 2829-516 Caparica, Portugal; 3 Human Immunobiology and Pathogenesis, CEDOC-Chronic Diseases Research Centre, NOVA Medical School, Faculdade de Ciências Médicas, Universidade Nova de Lisboa, 1150-082 Lisbon, Portugal; helena.soares@nms.unl.pt; 4 Experimental Pathology and Therapeutics Group, Portuguese Institute of Oncology, 4200-162 Porto, Portugal; jose.a.ferreira@ipoporto.min-saude.pt; 5 Porto Comprehensive Cancer Center (P.ccc), 4200-072 Porto, Portugal; 6 Center for Proteomics and Metabolomics, Leiden University Medical Center, 2300 RC Leiden, The Netherlands; P.J.Hensbergen@lumc.nl (P.J.H.); M.Pirro@lumc.nl (M.P.); 7 Amsterdam UMC, Vrije Universiteit Amsterdam, Department of Molecular Cell Biology and Immunology, Cancer Center Amsterdam, Amsterdam Infection and Immunity Institute, De Boelelaan 1117, 1081 HzAmsterdam, The Netherlands; s.vanvliet@vumc.nl; 8 Department of Life Sciences and Chemistry, Jacobs University, 28759 Bremen, Germany; s.springer@jacobs-university.de

**Keywords:** dendritic-cells, antigen-presentation, MHC-I, immunogenicity, T-cell response, cancer-vaccines

## Abstract

Maturation of human Dendritic Cells (DCs) is characterized by increased expression of antigen presentation molecules, and overall decreased levels of sialic acid at cell surface. Here, we aimed to identify sialylated proteins at DC surface and comprehend their role and modulation. Mass spectrometry analysis of DC’s proteins, pulled down by a sialic acid binding lectin, identified molecules of the major human histocompatibility complex class I (MHC-I), known as human leucocyte antigen (HLA). After desialylation, DCs showed significantly higher reactivity with antibodies specific for properly folded MHC-I-β2-microglobulin complex and for β2-microglobulin but showed significant lower reactivity with an antibody specific for free MHC-I heavy chain. Similar results for antibody reactivities were observed for TAP2-deficient lymphoblastoid T2 cells, which express HLA-A*02:01. Using fluorescent peptide specifically fitting the groove of HLA-A*02:01, instead of antibody staining, also showed higher peptide binding on desialylated cells, confirming higher surface expression of MHC-I complex. A decay assay showed that desialylation doubled the half-life of MHC-I molecules at cell surface in both DCs and T2 cells. The biological impact of DC´s desialylation was evaluated in co-cultures with autologous T cells, showing higher number and earlier immunological synapses, and consequent significantly increased production of IFN-γ by T cells. In summary, sialic acid content modulates the expression and stability of complex MHC-I, which may account for the improved DC-T synapses.

## 1. Introduction

Dendritic cells (DCs) are the most potent antigen-presenting cells, bridging the innate and adaptive immune responses [1,2]. T cell stimulation is essential for setting up immune responses, which are only achieved by proper DC maturation. The development of immunotherapies based on DCs has long resorted to ex vivo differentiation from the monocyte precursors [3,4], especially due to their relative high abundance in peripheral blood compared to DCs. DC-based anticancer vaccines production follows these steps: autologous CD14+ monocytes derived from peripheral blood of cancer patients are differentiated *in vitro* with GMCSF and IL-4 into immature DCs. After, immature DCs are loaded with tumor antigens and then further matured through cytokine based maturation cocktails [4,5]. Mature DCs are characterized by increased expression of antigen presentation molecules (e.g., Major Histocompatibility Complex (MHC) class-I and –II), co-stimulatory receptors (e.g., CD40, CD80, CD86) and cytokine production. The antigen presentation by DCs depends on their migration into lymph nodes, and on the establishment of an intricate cell-cell communication that culminates in the formation of the immunological synapse with T cells [6]. Cytotoxic CD8+ T cell engagement starts by DCs delivering the first signal through MHC-I. This early signaling step is considered to be of low affinity, as T cells simply “touch and go” DCs, unless a specific peptide presented via MHC molecules is recognized by the T cell receptor (TCR) [7,8]. Typically, MHC-I presents peptides generated by the degradation of endogenous proteins by the proteasome. These peptides are transported into the endoplasmic reticulum, where they are assembled with MHC-I heavy chain· complexed with β2 microglobulin (β2m) and the resulting peptide:MHC-I (pMHC-I) transit to the cell surface. The peptide or the β2m may then dissociate from the heterotrimer complex resulting in the appearance of free MHC-I heavy chain at the cell surface [9]. Free MHC-I heavy chains have reduced cell surface half-life, they do not activate T cells and they are typically internalized to enable the assembly with new peptides. Effector T cell activation is only triggered by the assembled heterotrimer and it can be evaluated by the secretion of interferon (IFN)-γ, a hallmark of T cell-mediated immune responses [10,11].

The cell surface of all immune cells is coated by a complex assortment of glycans, characteristically decorated at their terminal position by sialic acids. These sialic acids comprise a broad family of sugars derived from neuraminic acid, catalyzed by specific sialyltransferases. Due to their terminal position, sialic acid-containing glycoproteins are frequently recognized by lectin receptors, which include selectins and siglecs that modulate many biological processes such as cell signaling, cell-cell interactions and migration [12]. The cell surface of human DCs has a high content of α2,6-sialylated structures, due to the expression of ST6Gal-I sialyltransferases [13,14,15]. The levels of sialic acid at DCs surface were shown to modulate the maturation status of both murine and human DCs [16]. Previous results from our group showed that the temporary removal of surface sialic acids by sialidase treatment increased the ability of human monocyte-derived DCs to activate T cells and to provide antigen-specific anti-tumoral responses [17]. Desialylated DCs have higher expression of MHC-I, MHC-II and co-stimulatory molecules (e.g., CD80, CD86), and higher secretion of pro-inflammatory cytokines, which altogether contribute to a superior T_H_1 anti-tumoral response. ST6Gal-I knockout DCs also show increased capacity to activate T cells, further supporting that the ability of DCs to induce T_H_1 anti-tumoral responses is improved by sialic acid removal [16,17]. Initially thought as mere induction of DC maturation [16] the fact is that the molecular mechanisms behind this outcome are still poorly characterized.

In the present work, we have analyzed the molecular mechanism underlying sialic acid-induced modulation in DCs. We identified MHC-I as one of the major α2,6-sialylated molecules expressed in DCs. We showed that desialylated DCs have increased the number and stability of MHC-I heavy chain I-β2m complexes at the cell surface. At the molecular level, we propose that increased stability of MHC-I at the cell surface is due to differences in MHC-I turnover. We propose that there is a slower dissociation of MHC-I-β2m-peptide complexes, lower endocytosis, and lower degradation. The resulting increased stability of MHC-I complexes at the cell surface may contribute to their superior immunogenicity in eliciting CD8+ T cell-mediated responses. Our work paves the way to new biochemical investigations aiming to get further insights into how the removal of α2,6-sialic acids impacts the expression and regulation of MHC-I on human DCs, which can be exploited to develop anti-tumoral cell based-therapies.

## 2. Materials and Methods

### 2.1. Generation of Human DCs

Peripheral blood mononuclear cells (PBMCs) were isolated from buffy coats of healthy anonymous volunteers provided by Instituto Português do Sangue e da Transplantação (IPST), after written and informed donor consent IMP.74.52.4, according to the directive 2004/23/EC on setting standards of quality and safety for the donation, procurement, testing, processing, preservation, storage, and distribution of human tissues and cells (Portuguese Law 22/2007, June 29), with the approval of the ethics committee of IPST 30072015 Monocytes were selected using anti-CD14 coated immunomagnetic beads (Miltenyi Biotech, Germany), and differentiated for 5 days into DCs, using Roswell Park Memorial Institute (RPMI)-1640 medium, supplemented with 10% fetal bovine serum (FBS), 2 mM GlutaMAX, 1 mM sodium pyruvate, non-essential amino acids and 100 μg/mL penicillin/streptomycin, all from Gibco/Thermo Fisher Scientific (Waltham, MA, USA) in the presence of human recombinant GM-CSF (1000 U/mL) and IL-4 (750 U/mL) (Miltenyi Biotech, Germany) [17,18]. When appropriate, DCs maturation was induced after differentiation by the addition of 5 μg/mL of lipopolysaccharide (LPS) or 1000 U/mL of IL-1β, IFN-γ or TNF-α.

### 2.2. Cell Lines

The HLA-A*02:01 positive leukemia cell line T2 (ATCC^®^ CRL-1992™) was kindly provided by Professor Y. van Kooyk (VU University Medical Center, Amsterdam, The Netherlands). Cells were kept in culture using RPMI-1640 medium supplemented with 10% FBS, 2 mM GlutaMAX and 100 μg/mL penicillin/streptomycin. The colon cancer carcinoma cancer cell line SW948 (ATCC^®^ CCL-237™) was kindly provided by Professor Fabio Dall’Olio Department of Experimental Diagnostic and Specialty Medicine, University of Bologna, Bologna, Italy). Cells were cultured in Leibovitz’s L-15 medium supplemented with 10% FBS.

### 2.3. Sialidase Treatment

Enzymatic removal of sialic acid from the cell surface was performed using sialidase from Clostridium perfringens (Roche Diagnostics, Basel, Switzerland). Cells (5 × 106/mL) were treated using 100 mU sialidase, in RPMI-1640 media, for 60 min, at 37 °C. When appropriate, enzymatic removal of sialic acid from total cell lysates was performed with 20 mU of sialidase per 100 μg of total protein, in digestion buffer (50 mM sodium acetate, 5 mM CaCl2, pH 5.5), overnight at 37 °C.

### 2.4. Anti-MHC-I antibodies

Table 1 shows the list of monoclonal antibodies against major human histocompatibility complex class I (MHC-I) that were used in this study.

### 2.5. Flow Cytometry

Anti-MHC-I monoclonal antibodies are listed in Table 1 (see above). Mouse monoclonal hybridoma supernatants, HC10 and BBM.1, were used in a 1:4 dilution, and commercial antibodies were used following vendor instructions. For flow cytometry analysis, we also stained cells with FITC-conjugated Sambucus nigra (SNA) lectin (Vector Labs) and the fluorescently FITC-labeled peptides NLVPKFITCVATV and SIINFEKFITCL (Genecust). Cells were harvested and washed before staining with appropriate fluorophore-conjugated antibodies for flow cytometry. For peptide staining, a final concentration of 5 μM was used. Staining was performed at 4 °C for 30 min. Cells were then washed and fixed in 2% paraformaldehyde. Acquisition of data was performed on an Attune Acoustic Focusing Cytometer (Applied Biosystems, Waltham, MA, USA) and the FlowJo software version 10.0.5 (TreeStar, San Carlos, CA, USA) was used to analyze the mean fluorescent intensity (MFI) and cell percentages. The MFI represents the median fluorescence as calculated by the software. For each staining condition, the respective MFI of unstained/isotype control was subtracted.

### 2.6. Confocal Laser Scanning Microscopy

To perform confocal laser scanning microscopy, cells were plated on 12-mm diameter polylysine-coated glass coverslips and incubated for 5 min at room temperature. Coverslips were then centrifuged at 100 × g for 1 min to promote cell adhesion, fixed for 30 min with 4% paraformaldehyde (PFA) and washed using 1% bovine serum albumin (BSA) in PBS. Mouse anti-human HLA-ABC, clone 246-B8.E7 (see Table 1, above) was used for staining human MHC-I, followed by a fluorescently-conjugated secondary antibody. FITC-conjugated SNA lectin was also used to stain α2,6-linked sialic acids on the cell surface. Images were acquired on a Zeiss LSM710 confocal microscope (Zeiss, Oberkochen, Germany). Illustrative confocal cross-section pictures were selected after Z-stacking processing. Staining intensity was analytically quantified using the corrected total cell fluorescence (CTCF) = Integrated Density − (Area of selected cell × Mean fluorescence of background readings).

### 2.7. Protein Extraction, Immunoprecipitation, and Western Blotting

Whole cell lysates were obtained using Immunoprecipitation (IP) lysis buffer and the concentration of protein determined using the BCA Protein Assay Kit, all from Pierce, Thermo Fisher Scientific (Waltham, MA, USA). For protein affinity separation, an SNA-agarose column (EY Labs, San Mateo, CA, USA) was loaded with whole cell lysates. Unbound proteins were eluted using PBS and bound proteins were eluted using 0.1 M lactose in PBS. The collected bound protein fraction was concentrated using an Amicon Ultracel 3K column (Millipore) and both fractions were stored for further use.

IP of MHC-I protein were obtained from total protein extracts using clone W6/32 [16] (see Table 1, above) and Direct IP Kit from Pierce.

For Western blotting, protein samples were separated in SDS-PAGE gels, transferred to 0.45 μm nitrocellulose membrane (GE Healthcare Life Sciences, Piscataway, NJ, USA) and blocked with 7.5% non-fat milk. To detect MHC-I, an anti-HLA-ABC (W6/32) antibody and HC10 hybridoma supernatant were used as primary antibodies. In both cases, goat anti-mouse IgG1 heavy chain horseradish peroxidase (HRP) conjugate (Abcam, Cambridge, UK) was used as the secondary antibody. For lectin blotting, membranes were blocked overnight with 1% CarboFree (Vector Labs, Burlingame, CA, USA) and HRP conjugated-SNA lectin was used to probe the membrane. The Amersham ECL Prime Western Blotting Detection Reagent (GE Healthcare Life Sciences, Piscataway, NJ, USA) was used as a developing reagent. β-actin was used as endogenous control to quantify the relative amount of protein expression.

### 2.8. Mass Spectrometry and Bioinformatics

For glycoproteomics analysis, IP of MHC-I protein from DCs was separated on a 4–12% gradient SDS-PAGE gel (Thermo Fisher Scientific) under reducing conditions and subsequently stained with SimplyBlue Safe Stain (Thermo Fisher Scientific) and washed with distilled water. Protein bands of interest (between 38 and 49 kDa) were excised from the gel and proteins were subsequently in-gel digested with trypsin (Promega, WI, USA) after reduction (10 mM 1,4-dithiothreitol (Sigma Aldrich)) and alkylation (50 mM iodoacetamide (Sigma Aldrich).

For LC-MS/MS analysis, tryptic digests were separated by online C18 nano-HPLC MS/MS with an Easy nLC 1000 gradient HPLC system (Thermo, Bremen, Germany) coupled to an Orbitrap Fusion Lumos mass spectrometer (Thermo), as previously described [23]. Fractions were loaded onto a homemade precolumn (100 μm × 15 mm; Reprosil-Pur C18-AQ 3 μm, Dr. Maisch, Ammerbuch, Germany) and eluted via a homemade analytical nano-HPLC column (15 cm × 50 μm; Reprosil-Pur C18-AQ3 μm) with a gradient from 10% to 40% of solvent B (20/80/0.1 water/acetonitrile/FA *v*/*v*/*v*) for 20 min. The nano-HPLC column was drawn to a tip of ∼5 μm, which acted as the electrospray needle of the MS source. The Lumos mass spectrometer was operated in data-dependent Higher-energy Collisional Dissociation (HCD) MS/MS (top-10 mode) using at a normalized collision energy of 32% and recording of the MS2 spectrum in the Orbitrap. At the master scan (MS1) level, the resolution was 120,000 and the scan range was m/z 400−1500 at an AGC target of 400,000 with maximum injection time of 50 ms. Dynamic exclusion was applied after n = 1, with exclusion duration of 10 s, with mass tolerance of 10 ppm. For MS2, charge states 2−5 were included and precursors were isolated with the quadrupole with an isolation window of 1.2 Da. The MS2 scan resolution was 30,000 with an AGC target of 50,000 and maximum injection time of 60 ms.

For proteomics analysis, 10 μg of the protein pool was separated by 4–16% gradient SDS/PAGE (BioRad) under reducing conditions; the bands were excised from the gels, and proteins were reduced with 5 mM 1,4-dithiothreitol (Sigma Aldrich) for 40 min. at 60 °C, alkylated with 10 mM iodoacetamide (Sigma Aldrich) for 45 min. in the dark and digested with trypsin (Promega, WI, USA) overnight at 37 °C for mass spectrometry (MS) analysis. Protein identification was performed as previously described [24] Tryptic digests were separated with a C18 Pepmap (Dionex) column on an Ultimate 3000 (Dionex/LC Packings, Sunnyvale, CA) nano-HPLC, and fractions were collected with a Probot (Dionex/LC Packings, Sunnyvale, CA) directly onto a matrix-assisted laser desorption ionization (MALDI) plate. MS was performed on a 4800 MALDI-TOF/TOF Analyzer (Applied Biosystems, Foster City, CA). The MS and MS/MS spectra acquired were processed and analyzed by the Global Protein Server Workstation (Applied Biosystems). LC-MALDI-MS/MS runs were done in duplicates. Protein identification was achieved with a search performed against the Swiss-Prot protein database (March 2009, 428,650 entries) for Homo sapiens. The final list includes proteins queried using the “Retrieve ID/mapping” tool of UniProtKB for membrane glycoproteins with extracellular domain prone to exhibit or secreted glycoproteins, as previously described [25]. The presence of glycosylation sites was confirmed in silico with NetNglyc 1.0 server, an artificial neural network that examines the sequence context of Asn-X-Ser/Thr (where X is not Pro) sequons [26], and with NetOglyc 4.0 server, that produces neural network predictions of mucin-type GalNAc O-glycosylation sites in mammalian proteins [27]. Glycoproteins biological and molecular functions were annotated based on gene ontology (GO) terms using STRAP 1.5 (Software Tool for Rapid Annotation of Proteins: Cardiovascular Proteomics Center, Boston University School of Medicine, Boston, MA, USA).

### 2.9. Peptide Stability Assays

For peptide stability binding assays, T2 cells were incubated with HLA-A*02:01 matching peptides (YLEPGPVTA from gp100 or NLVPMVATV from CMVpp65 antigens) in concentrations ranging from 1 to 100 μM, and 10 μg/mL of β2m (Sigma-Aldrich, St. Louis, MO, USA), in RPMI-1640 medium. Controls without peptide were performed in parallel. The sialic acid removal was performed with 100 mU sialidase for 1 h at 37 °C immediately before peptide incubation. For sialic acid addition other experiments, cells were incubated in the same conditions, with 2.1 mU recombinant human ST6Gal-I and 50 μM CMP-sialic acid (Sigma-Aldrich), instead of sialidase. Recombinant ST6Gal-I was a kind gift from Professor Joseph Lau (Roswell Park Comprehensive Cancer Center, Buffalo, NY, USA), prepared as described elsewhere [28]. After 3 h, cells were harvested and analyzed by flow cytometry for the expression of MHC-I, using an anti-HLA-A2 antibody (BB7.2).

### 2.10. Surface MHC-I Stability Assays

DCs or T2 cells were treated with 5 μg/mL of the Golgi-export inhibitor Brefeldin A (BFA) (Sigma-Aldrich) and incubated at 37 °C, in the presence of RPMI-1640 medium, for 5 h. When treating cells with sialidase, 100 mU of this enzyme was added simultaneously with BFA. After 1 h, the sialidase was removed by centrifugation and the cells were plated again with RPMI-1640 supplemented with 5 μg/mL BFA during the course of the experiments. At each time point, cells were harvested and the cell surface expression of MHC-I molecules was assessed by staining cells with an anti-HLA-A2 antibody (BB7.2) and analyzed by flow cytometry.

### 2.11. Gene Expression Assays

Total RNA was extracted from 1–5 × 106 DCs or T2 cells using the NZY Total RNA Isolation kit (NZYTech, Lisbon, Portugal). Genomic DNA was removed using the RNase-Free DNase Set (Qiagen, Germany), following manufacturers’ instructions. RNA concentration from each sample was determined spectrophotometrically and then reverse transcribed into cDNA using random primers and the High Capacity cDNA Reverse Transcription Kit (Applied Biosystems, Foster City, CA).

Real-time PCR was performed in a Rotor-Gene 6000 (Corbett Life Science, Sydney, Australia) using TaqMan^®^ Fast Universal PCR Master Mix. The assays containing both TaqMan^®^ probes and primers (Thermo Fisher Scientific, Waltham, MA, USA) for a specific gene were: HLA-A (human leukocyte antigen A allele; Hs01058806_g1), β-actin (4352935E) and GAPDH (glyceraldehyde 3-phosphate dehydrogenase; 4333764F) were used. Each reaction was performed in duplicate.

Messenger RNA (mRNA) expression was normalized using the geometric mean of the expression of two endogenous controls, β-actin and GAPDH, as a reference. The relative expression of each gene was calculated using the cycle threshold (Ct) method and compared accordingly to the 2−ΔΔCt equation [29]. The efficiency of the amplification reaction for each primer/probe was above 95% (as determined by the manufacturer).

### 2.12. DC: T Cell Interaction/ Doublet Analysis

For DC: T cell doublet analysis, DCs were generated as previously described and autologous CD8+ T cells were isolated by immunomagnetic separation using anti-human CD8 beads (Miltenyi, Germany). DCs were fluorescently labeled with CellTrace™ Far Red (Thermo Fisher) and CD8+ T cells were fluorescently labeled with CellTrace™ carboxyfluorescein succinimidyl ester (CFSE, Thermo Fisher) and co-cultured (1.1 × 10^6^ cells/mL) in a 96-well plate. The ratio of DCs to T cells was 1:10 in a total volume of 200 μL of RPMI-1640 medium. After 6 or 24 h of co-culture, cells were collected, gently pippeted and analyzed using an Attune Acoustic Focusing Cytometer (Applied Biosystems, USA). The gating strategy excluded single cells, and DC: T cell doublets were positively identified when the two fluorescences were simultaneously co-localized.

### 2.13. Cytokine Production Evaluation

To evaluate intracellular cytokine production, DCs were co-cultured with autologous CD8+ T cells (1:10 ratio) for 24 or 48 h, as described in the previous section. BFA was used to block cytokine secretion for the last 4 h of co-culture within the referred period. Cells were then fixed and permeabilized using the BD Cytofix/CytopermTM kit (BD Pharmingen, CA, USA). Cell staining was performed using a PE-conjugated anti-human IFN-γ antibody, clone B27 (Biolegend) and a PerCP-conjugated anti-human CD8 antibody, clone HIT8a (Immunotools, Germany). After washing steps, cells were analyzed by flow cytometry. Untreated DCs were used as control.

### 2.14. Statistical Analysis

Statistical analysis was performed using the GraphPad Prism 6.0 software (GraphPad Software, La Jolla, CA, USA). Unless otherwise stated, statistical significance (*p* value) was calculated using the two-tailed paired t-test. Statistical significance was defined as *p* < 0.05 (*), *p* < 0.01 (**) and *p* < 0.001 (***).

## 3. Results

### 3.1. MHC Class I Molecules on Human Dcs are Sialylated

**Figure 1:**
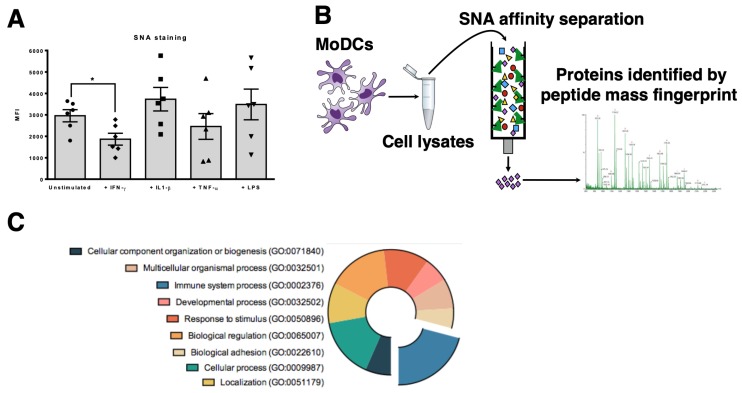
Mass spectrometry analysis of SNA-binding proteins isolated from DCs reveals key immune-related proteins. (**A**) The reactivity of *Sambucus nigra* (SNA) lectin to α2,6-sialylated glycans on the surface of human DCs was quantified by flow cytometry after different maturation stimuli: IFN-γ, IL-1β, TNF-α and LPS. Unstimulated DCs were used as control. Values presented are mean ± SEM (N = 6). Statistically significant differences are indicated by asterisks (* *p* ≤ 0.05). (**B**) Schematic representation of the steps followed to identify α2,6-sialylated proteins from DCs. Whole cell lysates of human DCs were immunoprecipitated through a SNA-binding column. The eluted proteins were analyzed by mass spectrometry and the corresponding identified scores were matched and associated with Gene Ontology (GO) entries. (**C**) Distribution of the identified sialylated proteins by their molecular function. Pie chart represents different molecular functions of the identified proteins, according to the GO entries. Immune system processes were highlighted.

The expression of α2,6-sialic acids by human DCs was evaluated by the reactivity with SNA lectin, which preferentially recognizes α2,6-linked sialic acids. Data showed that DCs are highly α2,6 sialylated. During maturation, the α2,6 sialylation pattern is altered, decreasing significantly with IFN-γ and being only slightly modulated with the pro-inflammatory stimulus IL-1β, TNF-α and LPS (Figure 1A). The data is in agreement with previous reports [13,14] and suggests that when surface sialylated proteins change their sialic acid content, they also change their behavior. To identify α2,6-sialylated proteins expressed by DCs, we immunoprecipitated proteins from total DCs lysates using the SNA lectin. Isolated SNA-binding proteins were identified by Mass Spectrometry (Figure 1B and Supplementary Figure S1) and the proteins with higher scores (Table S1) were matched, associated by Gene Ontology and sorted accordingly to different biochemical functions. Considering only membrane-associated proteins, the main cellular function of the eluted fraction was related to immune system processes (GO: 0002376) (26% of all identified proteins) (Figure 1C). The protein with higher score sequence coverage were alleles of the HLA-A molecules, followed by HLA-B and HLA-C alleles, all known for their high degree of polymorphism and sequence homology (Table S1).

**Figure 2:**
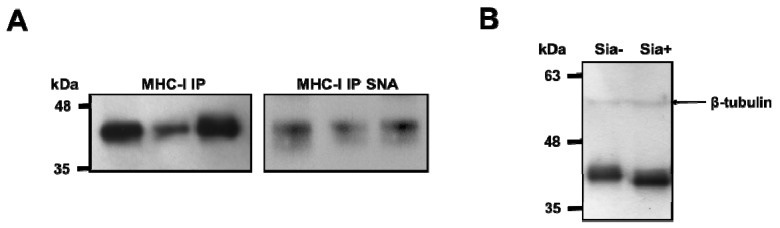
MHC-I on human DCs is sialylated. (**A**) MHC-I immunoprecipitates (IP) show reactivity with SNA lectin. MHC class I immunoprecipitates were obtained from whole cell lysates of DCs from 3 different individuals IP products were probed with anti-MHC-I (W6/32 antibody) (left) and SNA lectin to confirm the presence of α2,6-linked sialic acids (right) (**B**) Molecular weight of MHC-I heavy chain is decreased after sialidase treatment. Whole cell lysates of human DCs were treated with sialidase (Sia+) overnight at 37 °C and separated by SDS-PAGE and then probed for anti-MHC class I, using the W6/32 antibody in Western blot. Untreated cell lysates (Sia-) were used as control and β-tubulin was used as loading control.

To evaluate the sialylation of MHC-I molecules, these were immunoprecipitated from DCs of different individuals and probed with SNA lectin on Western blot. Immunoprecipitates showed a strong SNA binding, demonstrating the presence of α2,6-sialylated structures on MHC class I heavy chains (Figure 2A). To further confirm that MHC-I molecules in DCs were sialylated, whole cell lysates were treated with sialidase, electrophoresed and stained with an anti-HLA-ABC antibody W6/32 (see Table 1) on Western blot (Figure 2B). The resulting blots showed a band with a molecular weight of approximately 40–45 kDa, similar to the predicted molecular weight of the MHC-I heavy chain; the molecular weight of this band was decreased when proteins were desialylated, suggesting that MHC-I heavy chain is decorated with sialic acid (Figure 2B). Decreased molecular weight of the MHC-I heavy chain after sialidase treatment of whole cell lysate was also confirmed with the antibody HC10, which is specific for the free MHC-I heavy chain (Supplementary Figure S2). These results suggest that α2,6-sialylation is a common feature among different heavy chain alleles of MHC-I.

To confirm that HLA-A is sialylated we analyzed the MHC-I immunoprecipitates from DCs by LC-ESI-MS/MS. The N-glycosylation site Asn110 was occupied by mono and di-sialylated glycan structures. For example, the triply charged ion (M+3H)3 + at m/z 1252.8385 (Supplementary Figure S3) correspond to the tryptic peptide GYYNQSEAGSHTVQR decorated with a bi-antennary mono-sialylated N-glycan structure and the triply charged ion at m/z 1349.8696 (M+3H)3+ correspond to the above-mentioned peptide decorated with a bi-antennary di-sialylated N-glycan structure (data not shown). Therefore, the glycopeptide analysis confirmed the presence of sialylated N-glycans on Asn110 of HLA-A.

### 3.2. Desialylation Increases the Number of Mhc Class I-Β2m Dimer Complex in Dcs and T2 Cell Line

**Figure 3:**
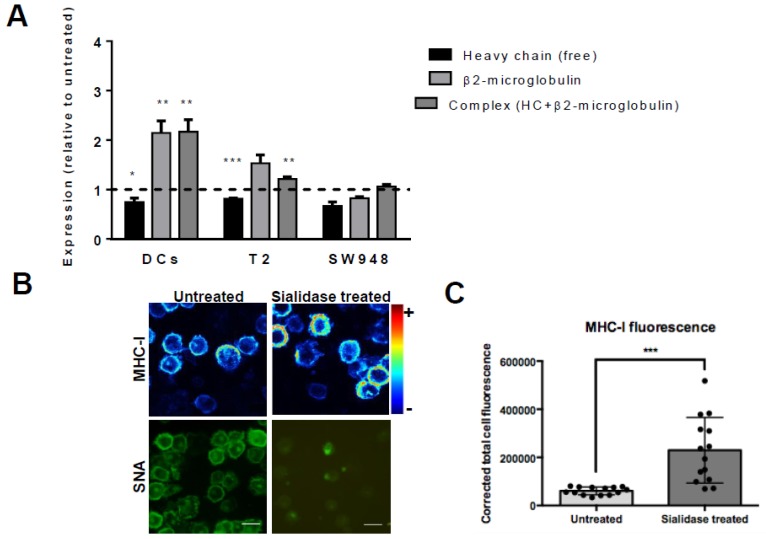
Desialylation increases MHC class I molecules in DCs and T2 cell line but not in the SW948 colon cancer cell line. (**A**) DCs, T2 cell line, and SW948 cell line were treated with sialidase for 1 h at 37 °C and analyzed by flow cytometry for MHC-I staining (using the HC10 (free heavy chain); β2m (BBM.1 antibody) and the complex heavy chain+β2m (BB7.2 for T2 and W6/32 antibody for MoDCs and SW948) (**B**) Confocal microscopy pictures of immature human DCs probed for surface staining of MHC-I (W6/32 antibody). Human DCs were treated with sialidase and prepared on coverslips for confocal microscopy. A range of z-stack images was collected from different cells and processed to include mean staining intensity. Scale bar equals 20 µm. (**C**) The fluorescence of MHC-I by confocal microscopy was quantified accordingly to the corrected total cell fluorescence (CTCF) method on at least 14 different cells per slide, as described in the Methods section. Values presented are mean ± SEM (N ≥ 5). Statistically significant differences are indicated by asterisks (* *p* ≤ 0.05, ** *p* ≤ 0.01, *** *p* ≤ 0.001).

To assess if there is an inverse relationship between the content of cell surface sialic acids and expression of MHC-I, we analyzed the expression of different MHC-I molecular assemblies/conformations at cell surface right after sialidase treatment. For that purpose, we have used monoclonal antibodies that bind to different MHC-I assemblies/conformations (see Table 1) and also different cells. Besides human DCs, we used T2 cells, TAP2-deficient lymphoblastoid cells which express low levels of HLA-A02*01 at cell surface due to lack of endogenously bound peptides, and the epithelial colon cancer cell line SW948. The W6/32 antibody recognizes amino acids of the HLA-A,B,C chain in their three-dimensional configuration, which is stably maintained when the HLA-A,B,C heavy chain is associated with β_2_m [30], whereas the BB7.2 antibody, is also a conformation-specific antibody that binds only HLA-A2 (see Table 1). Therefore, to detect the presence of properly folded MHC-I heavy chain-β2m dimer complex at cell surface we have used W6/32 to stain DCs and SW948 cells and the BB7.2 antibody to stain T2 cell line and analyzed by flow cytometry. To further validate the results we have used the antibody HC10, which binds only the free MHC-I heavy chain, but not to MHC-I heavy chain-β2m dimer complex [20] and BBM.1 which bind to β2m either free or associated to MHC-I heavy chain [21]. By flow cytometry, in DCs and T2 we observed a remarkable significant increase in the staining of W6/32 (2.17 ± 0.24 times) and BB7.2 (1.2 ± 0.47 times), respectively, in sialidase treated as compared with untreated cells (Figure 3A). When we stained with BBM.1, which binds only to β2m, we observed a parallel significant higher staining after sialidase treatment (2.15 ± 0.24, for DCs and 1.50 ± 0.17, for T2). Conversely, the staining of the free heavy chain with HC10 decreases (0.75 ± 0.08, for DCs and 0.80 ± 0.015, for T2). These results indicate that for both DCs and T2 cells, sialidase treatment increases the number of MHC-I heavy chain- β2m complexes, while decreasing the number of free heavy chains at cell surface. On the other hand, for the cancer cell line SW948 there were no significant differences in the staining for any of the antibodies for MHC-I molecules, demonstrating that the increase in MHC-I levels induced by sialidase removal seems to be restricted to leucocytes (Figure 3A). The efficient enzymatic removal of sialic acids from the cell surface was always confirmed by lectin staining and flow cytometry analysis (results not shown).

To further investigate the influence of sialic acid removal on the distribution of MHC-I molecules at the cell surface, DCs were stained with 246-B8.E7, which binds to MHC-I heavy chain-β2m complex (see Table 1) and imaged by confocal microscopy after sialidase treatment. The expression of MHC-I molecules increased homogenously at cell surface after sialic acid removal, while SNA lectin binding is nearly absent (Figure 3B). Image processing with matching staining intensity showed significantly increased fluorescence of MHC-I molecules on sialidase-treated cells. In fact, there was a statistically significant increase of MHC-I CTCF on desialylated human DCs, compared with untreated cells (CTCF 229,699 ± 136,268 vs. 60,644 ± 16,127) (Figure 3C).

### 3.3. MHC Class I Molecules Show Increased Peptide Stability at the Cell Surface after Desialylation

**Figure 4:**
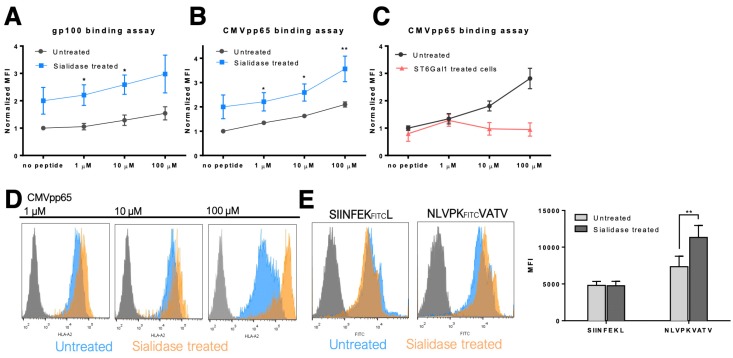
MHC class I molecules stability at cell surface after desialylation. T2 cells were used to study MHC-I stability after sialic acid removal from cell membrane. T2 cells were incubated with either (**A**) gp100 peptides or (**B**) CMVpp65 peptides, which match the HLA-A*02:01 pocket and stabilize the peptide-MHC-I-β2 complex. Cells were then probed with anti-HLA-A*02 antibody (BB7.2) and analyzed by flow cytometry. (**C**) T2 cells were extrinsically sialylated with a recombinant ST6Gal-I enzyme and incubated with the CMVpp65 peptide. Cells were then probed by flow cytometry for HLA-A*02 (using the BB7.2 antibody) expression at the cell surface. Graphs show normalized mean fluorescent intensity (MFI) values of at least 4 independent experiments. (**D**) Representative flow cytometry histograms of T2 cells untreated (in blue) or after sialidase treatment (in orange) and incubated with different concentrations of CMVpp65 peptide. (**E**) The fluorescently labeled NLVPK_FITC_VATV peptide was used to stain HLA-A2 on desialylated T2 cells and the fluorescently labeled SIINFEK_FITC_L peptide was used as irrelevant control. The quantification of the mean fluorescence intensity of fluorescently labeled peptides presented by T2 cells is also presented. Values presented are mean ± SD of at least N = 4 independent experiments. Statistically significant differences are indicated by asterisks (* *p* ≤ 0.05, ** *p* ≤ 0.01).

To investigate the mechanisms that regulate the immediate increase in MHC-I expression at the DCs surface, after sialidase treatment, a TAP-deficient cell line (T2) was used to study MHC-I stability. T2 cells are HLA-A*02:01 positive cells, expressing low levels of this MHC-I allele at cell surface due to their inability to efficiently load peptides into the endoplasmic reticulum (ER), hindering the formation of stable peptide:MHC-I:β2m complexes [31]. In the presence of peptides that match the MHC-I heavy chain pocket fold, the complexes of MHC-I and β2m are stabilized, resulting in increased expression at the cell surface of T2 cells. To assess the effect of desialylation, the CMVpp65 (NLVPMVATV) and gp100 (YLEPGPVTA) peptides, which match the HLA-A*02:01 pocket [32,33] were used in T2 cells treated or not treated with sialidase. As shown in Figure 4, sialidase treated T2 cells show a general higher staining of HLA-A*A02 molecules at the cell surface, when incubated with either peptide. For the gp100 peptide experimental assay, a statistically significant increase in HLA-A*A02 molecules was observed when desialylated T2 cells were incubated with peptide concentrations of 1 μM (MFI fold increase 2.21 ± 0.38 vs. 1.05 ± 0.06) and 10 μM (MFI fold increase 2.59 ± 0.35 vs. 1.29 ± 0.10), compared to untreated cells (Figure 4A). Similarly, desialylated T2 cells incubated with CMVpp65 peptide, also showed a significant increase in HLA-A*A02 molecules at the cell surface (Figure 4B,D) for all peptide concentrations. Additional experiments using the recombinant sialyltransferase ST6Gal-I for extrinsic α2,6-sialylation of T2 cells followed by incubation with the CMVpp65 peptide showed reduced expression of MHC-I at the cell surface after treatment (Figure 4C).

These results suggest that the stability of HLA-A*A02-peptide complexes on the cell membrane is influenced by cell surface sialylation. To exclude the possibility that desialylation lead to an increased anti-MHC-I antibody binding affinity (reactivity), we used a fluorescently labeled peptide, the CMVpp65 peptide NLVPKFITCVATV, that matches and binds to the HLA-A*A02 pocket to assess surface MHC-I expression. When incubating desialylated T2 cells with fluorescently labeled CMVpp65 peptide, we could observe a significant increase of peptide presentation via MHC-I (MFI 10,560 ± 142 vs. 7586 ± 130), compared to untreated cells. This increase was not observed using an irrelevant (SIINFEKFITCL) peptide. These results are aligned with the flow cytometric quantification and confirm that desialylation increases the expression of MHC-I molecules at the cell surface. The increased expression after desialylation indicates that MHC-I is a potential molecular target for sialidase enzymes.

### 3.4. MHC Class I Molecules Show Slower Turnover after Desialylation

**Figure 5:**
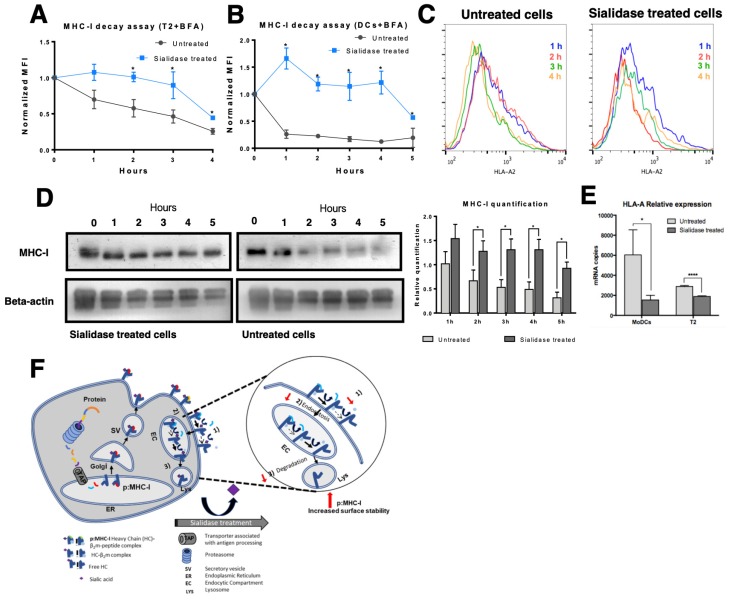
Effect of sialic acid removal on MHC class I molecules internalization. Cells were incubated with Brefeldin A (BFA) and treated with sialidase for 1 h, after which sialidase was removed but BFA remained for the course of the experiment. The cell surface expression of MHC-I molecules was assessed over time by staining cells with an anti-HLA-A*02 antibody (BB7.2) and analyzed by flow cytometry in (**A**) T2 cells (N = 3) or (**B**) immature DCs (N = 5). (**C**) Representative histograms of antibody staining of untreated and sialidase treated human DCs over time (from 1 h to 4 h). (**D**) Whole cell lysates of sialidase treated and untreated DCs were obtained at each time point and probed for MHC-I (W6/32) and β-actin (loading control) by Western blot. Lysates from each experimental setup (untreated vs. sialidase treated cells) were loaded into different gels and processed in parallel. The relative quantification of MHC-I expression was calculated comparing to the expression of β-actin (N = 5). (**E**) HLA-A gene expression was assessed by qPCR following sialidase treatment on DCs (N = 7) and T2 cells (N = 3). (**F**) Schematic representation of the suggested effect of sialidase treatment on the molecular events that occur during MHC-I turnover. After synthesis and assembly in the ER, peptide: MHC-I complex (pMHC-I) is formed by a heavy chain, non-covalently bound to β2m chain and a specific peptide. In pMHC-I, the heavy chain is further glycosylated in the Golgi and then exported to cell surface. Part of the MHC-I molecules that are endocytosed is recycled back to the cell surface, while others are degraded. Upon extrinsic desialylation, we suggest that there is a slower dissociation of β2m chain and of specific peptide, probably slowing internalization by endocytose and decreasing degradation. Values presented as mean ± SEM. Statistically significant differences are indicated by asterisks (* *p* ≤ 0.05, ** *p* ≤ 0.01, *** *p* ≤ 0.001).

While results from the previous section suggest that the stability of MHC-I at the cell surface is improved after desialylation, they do not exclude the possibility that increased protein export and incorporation into cell membrane may also be responsible for the increased cell surface levels of MHC-I, after desialylation. To assess this, we blocked the export of nascent glycoproteins to the cell surface on both T2 cells and HLA-A*A02^+^ DCs during sialidase treatment. For blocking protein export, we used BFA, which disrupts the Golgi apparatus [34], preventing MHC-I molecules and other proteins from being exported to the cell surface. Cell surface HLA-A*A02 molecules were then analyzed by flow cytometry at defined time points after treatment, as a readout indicative of internalization kinetics (turnover) or decay rate (membrane stability) (Figure 5). As shown in Figure 5A,C, in T2 cells the decay or internalization rate of desialylated HLA-A*A02 molecules is remarkably slower than non-treated cells. Namely, 2 h after treatment the HLA-A*A02 levels remain approximately like the initial value. In contrast, considering the same time point, fully sialylated T2 cells cultured in the presence of BFA lose approximately 50% of their surface HLA-A*A02 molecules, with an estimated half-life of *t*_1/2_ ~ 3 h.

In DCs, the decay or internalization rate of desialylated HLA-A*A02 molecules is also remarkably slower than in non-treated cells. However, DCs appear to have different HLA-A*A02 turnover kinetics, under the same experimental conditions (Figure 5B). Right after sialidase treatment (1 h), in the presence of BFA, DCs show a 1.5-fold increase in the content of HLA-A*A02 at the cell surface. In contrast, fully sialylated cells lose approximately 50% of cell surface MHC-I after 1 h indicating that the half-life of MHC-I molecules on fully sialylated DCs is *t*_1/2_ ~ 1 h.

To assess the total MHC-I content of the cells submitted to protein export blockade with BFA, whole-cell lysates of DCs were prepared at each time point and probed with an anti-HLA-ABC antibody on Western blot. As shown in Figure 5D, on desialylated DCs, the total amount of MHC-I protein remained constant during the entire course of the experiment. These results suggest that even though by flow cytometry, we observed an initial 1.5-fold increase in the membrane expression of HLA-A2, the total amount of MHC-I in the cell remained unaltered on desialylated cells. These results contrast to the amounts of MHC-I protein on untreated cells, where MHC-I is significantly degraded readily over time (Figure 5D), evidencing reduced protein stability. On untreated cells, the membrane expression of HLA-A2 approaches 50% of initial expression level, as measured by flow cytometry (Figure 5B), which is concordant with the total MHC-I expression observed by Western blot (Figure 5D).

We also found evidence that total MHC-I is not upregulated on cell membrane due to increased gene expression, following desialylation of cell surface. Analysis of *HLA-A* expression by qPCR shows a statistically significant reduction in gene expression on both DCs and T2 desialylated cells, compared to respective untreated cells (Figure 5E). In sialidase treated DCs, *HLA-A* showed a 4-fold decreased gene expression (mRNA copies 1540 ± 468 vs. 6044 ± 2499 for untreated cells). In T2 cells, which have a lower basal expression level of MHC-I molecules, desialylated cells showed a nearly 2-fold reduced *HLA-A* gene expression (mRNA copies 1895 ± 61 vs. 2889 ± 95 for untreated cells) (Figure 5E). Gene expression analysis indicates that increased MHC-I molecules seen at cell surface after desialylation are not due to increased gene expression.

Altogether, our results indicate that the membrane stability and the half-life of MHC-I molecules are modulated by their intrinsic sialic acid content, suggesting that pMHC-I complexes are more stable on the cell surface when the heavy chain is desialylated. As shown in Figure 5F after synthesis and assembly in the ERpMHC-I is further glycosylated in the Golgi and exported to cell surface. The half-life of this complex at the cell surface varies for different cell types. There is a dynamic exchange between MHC-I molecules at the surface and intracellular endocytosis compartments. Loss/dissociation of the β2m chain and of specific peptide can occur either in the cell surface or endocytic compartment. Part of the MHC-I molecules that are endocytosed is recycled back to the cell surface, while others are degraded [35]. We suggest that when desialylated there is a slower dissociation of β2m and/or specific peptide from the heavy chain, lower internalization by endocytose and lower degradation.

### 3.5. DC:T Cell Doublet Formation and T Cell Activation are Improved by Desialylated DCs

**Figure 6:**
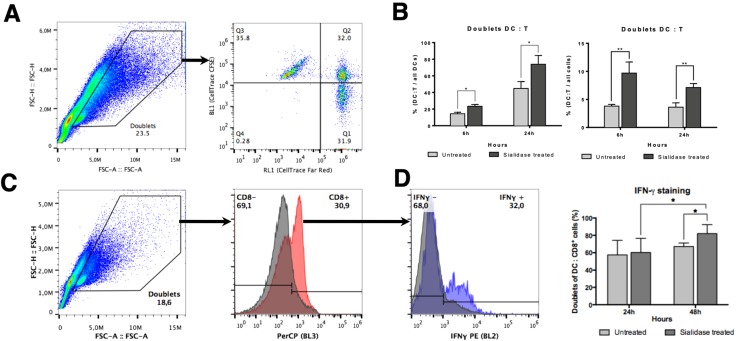
Co-cultures of DCs (untreated or sialidase treated) with autologous CD8^+^ T cells. (**A**) Gating strategy used to identify DC:T cell interactions. Co-cultures of FarRed^®^-labeled DCs with CFSE-labeled autologous CD8^+^ T cells were analyzed by flow cytometry. Single cells were excluded and doublet identification was considered when the two fluorescences co-localize. (**B**) Percentage of DC:T cell doublets in relation to all DCs.. The number of doublet DC:T cell and of the number of total DCs (as evaluated by staining with FarRed fluorescence) was quantified by flow cytometry at 6 h and 24 h of co-culture. The graph represents the percentage of DC:T cell doublets in relation to the total number of DCs (N = 6). (**C**) Percentage of DC:T cell doublets in relation to all cells.. The number of doublet DC:T cell and of the number of total events was quantified by flow cytometry at 6 h and 24 h of co-culture. The graph represents the percentage of DC:T cell doublets in relation to the total number of cells (doublet and non-doublet) (N = 6). (**D**) Intracellular IFN-γ production was evaluated at 24 h and 48 h of co-culture. The left panel shows the gating strategy, which considers doublet populations of DC:T cells stained with the anti-human CD8 (HIT8a) antibody and with anti-IFN-γ (B27). The right panel shows a graph representing the percentage of CD8^+^ cells that express IFN-γ in relation to the total number of DC:T cells interacting (N = 5). Values presented as mean ± SEM. Statistically significant differences are indicated by asterisks (* *p* ≤ 0.05, ** *p* ≤ 0.01).

The ability of DCs to prime T cells requires activation signals that include the recognition of specific MHC-I-bound peptides by the TCR. The DC:T cell synapse promotes TCR clustering and sustained signaling. A sustained TCR signaling essential to promote T cell activation requires at least 20 h of contact between DC and T cell [36], which can be reduced in the presence of co-stimulation [37]. Since desialylated DCs have a higher content of cell surface MHC-I and co-stimulatory molecules on their surface [16,17], we reasoned that this may increase their ability to interact with T cells and prime them, promoting antigen-specific cytotoxic responses. Thus, we assessed the level of interaction between desialylated DCs and autologous CD8^+^ T cells and verified if these interactions would lead to a functional T cell activation. For that purpose, co-cultures of fluorescently labeled DCs and autologous CD8^+^ T cells were established and a doublet analysis by flow cytometry was performed as a measure to assess conjugates/interactions between DCs and T cells (Figure 6A). The gating strategy included the selection of doublet events and considered a DC:T cell positive interaction when both fluorescences were detected on the same event by flow cytometry. As shown in Figure 6B, when DCs are desialylated, the percentage of DCs that establish interactions with CD8^+^ T cells (i.e. the doublets) in relation to all DCs detected is significantly higher at 6 h (% 23.3 ± 2.2 vs. 14.4 ± 1.8 for untreated DCs) and 24 h (% 73.9 ± 10.6 vs. 44.9 ± 8.2 for untreated DCs). In relation to all cells, the percentage of DC:T cell interactions are also significantly higher when DCs are previously desialylated at 6 h (% cells 9.6 ± 1.9 vs. 3.8 ± 0.3 for untreated cells) and 24 h (% cells 7.1 ± 0.7 vs. 3.6 ± 0.8) (Figure 6C). We also calculated the population formed by doublets DC:T cell within the total of population that formed doublets. Similarly, desialylated DCs show a statistically significantly higher ability to form doublets with autologous CD8^+^ T cells either after 6 h (% of doublets 77.3 ± 5.7 vs. 59.5 ± 5.2 for untreated cells) or 24 h in co-culture (% of doublets 89.8 ± 3.4 vs. 75.6 ± 3.1 for untreated cells) (Supplementary Figure S4A). Interestingly, the number of DC:DC doublets significantly decreases in relation to the total of doublets, when DCs were desialylated (Supplementary Figure S4B). Considering that the immunological synapse is stable for many hours, the results suggests that desialylation increases the number of specific interactions between DC and CD8^+^ T cells, strengthening over time. When determining the MFI of the fluorescence attributed to T cells (CFSE)-within DC:T cell doublets, a higher fluorescence is observed for desialylated DCs (Supplementary Figure S4C). These results suggest that each desialylated DCs interacts with more CD8^+^ T cells than untreated DCs.

The production of cytokines is one of the hallmarks of T cell activation. While IL-2 remains the most important cytokine driving the autocrine T cell expansion and activation, we sought to assess the intracellular production of IFN-γ, the major T_H_1 cytokine involved in anti-viral and anti-tumoral responses, upon the establishment of the immunological synapse with desialylated DCs. After 24 h in co-culture, the intracellular levels of IFN-γ were similar on T cells primed with fully sialylated or desialylated DCs (Figure 6D). However, co-culturing these cells for 48 h elicits a statistically significant increase in the intracellular staining of IFN-γ (% of doublet CD8+ T cells 82.0 ± 4.7 vs. 67.2 ± 1.8 for untreated DCs), where there are more CD8+ T cells becoming activated by interaction with desialylated DCs. Moreover, we found evidence that the expression of IFN-γ is dependent on the conjugate formation (data not shown). The population of single T cells expressing intracellular IFN-γ is lower, even when co-cultured with desialylated DCs. This result suggests a relationship between conjugate formation and IFN-γ production, which is improved by desialylated DCs.

## 4. Discussion

Nearly all cell surface proteins involved in antigen presentation and T cell activation, bear at some point sialylated glycans and are potential targets for the enzymatic removal of sialic acids on the cell surface. Cell surface receptors whose expression or signaling was already shown to be altered, following desialylation, include co-stimulation molecules (e.g., CD80, CD86) and innate immunity receptors [16,38]. Indeed, the activity of innate immune receptors (e.g., TLR-2, -3 and -4) is dependent on and potentiated by the activity of intrinsic sialidases [38,39]. Similarly, increased immune cell interaction with extracellular matrix components (e.g., ICAM-1), is associated with the activity of cell membrane-bound sialidases and lower levels of sialic acids [40].

MHC-I is expressed by all nucleated cells and plays a key role in driving antigen-specific immune responses by presenting peptides to CD8^+^ T cells. As virtually all cell surface proteins are glycosylated, MHC-I molecules were also described to include highly conserved glycosylation sites [41] In humans, the consensual glycosylation site is located on the heavy chain in the loop connecting the α1 and α2 domains, which form the binding groove for peptide presentation [42] However, proper glycan identification and characterization of this glycoprotein in human DCs is still lacking. Our results showed that the expression level of MHC-I at the cell surface is sensitive to the enzymatic removal of sialic acids, and SNA lectin reactivity against MHC-I molecules immunoprecipitated using a pan-HLA-ABC class I antibody (W6/32) suggests that sialylation is a common feature of different class I alleles. It also suggests that the degree of sialylation can differ between different alleles and may have a role in differential peptide presentation. The degree of sialylation of a specific allele may modulate the immune response, mainly by glycan steric effects, since the binding pocket (fold) for peptide presentation is in close proximity to the glycan chain [43].

Immature human DCs, which typically have lower expression of MHC-I than mature DCs, are known to be highly sialylated [16]. Flow cytometry analysis of DCs after sialidase treatment showed a statistically significant upregulation of MHC-I signal at the cell surface, both on T2 cells and DCs. Even though target protein glycosylation can affect antibody binding to certain epitopes [44,45], improved MHC-I signal due to increased antibody affinity and staining against desialylated cells seems implausible, since there was no improvement on MHC-I signal for desialylated SW948. (Figure 3A). Moreover, we used fluorescently labeled peptides, which are better presented via HLA*A02:01 on desialylated T2 cells, and also observed increased signal suggesting increased MHC-I molecules at the cell surface, which further validates our observations. When using gp100 and CMVpp65 peptides to stabilize desialylated MHC-I on T2 cells, we could not find significant differences in MHC-I MFI for the highest peptide concentration of gp100, between treated and untreated cells. This result suggests that gp100 and CMVpp65 peptides have different binding affinities for the MHC-I pocket. Using artificial neural networks to calculate peptide-MHC-I binding [46,47], the predicted affinity of gp100 peptide for the HLA-A02 pocket is 135.23 nM, in contrast with the higher binding affinity of the CMVpp65 peptide (for the same allele) of 25.85 nM. Considering this, we may infer that the stability of desialylated MHC-I molecules at the cell surface is also influenced, as expected, by the peptide affinity that stabilizes the pocket for presentation.

We hypothesized that the increased expression of MHC-I on desialylated DCs and T2 cells would be due to reduced internalization and degradation and higher membrane stability. To address this, we treated DCs and T2 cells with BFA, which blocks protein export from the Golgi apparatus but does not negatively affect the incorporation of recycling vesicles into cell membrane [48] Even though BFA is a well-known inhibitor of clathrin-mediated endocytosis, MHC-I turnover was already shown to be mediated by clathrin-independent endocytosis mechanisms [49,50]. Furthermore, although the small GTPases Arf1 and Arf5 are BFA sensitive it seems very unlikely that the traffic of MHC-I recycling vesicles and endosomes is affected since it is dependent on Arf6, which is not affected by BFA [51,52].

We have followed the expression of HLA-A*A02 at cell surface over time in a BFA decay assay. The untreated DCs lose approximately 50% of cell surface MHC-I after 1 h indicating that the half-life of MHC-I molecules on fully sialylated DCs is *t*_1/2_ ~ 1 h agreement with previous studies showing that MHC-I molecules have a short half-life (< 2 h) at the cell surface of DCs [35]. After desialylation, the initial 1.5-fold increase in the HLA-A*A02 expression may be explained either due to the presence of intracellular recycling vesicles, containing previously synthesized MHC-I molecules which are incorporated on cell membrane [49] by BFA-insensitive mechanisms, or other mechanisms regulating surface expression of membrane-bound proteins. Considering that in DCs there is evidence that approximately 50–60% of MHC-I molecules reside in recycling vesicles [35,49], our results suggest that desialylated MHC-I molecules, originating from recycling vesicles, are the main contributors to the increased surface expression of HLA-A2 molecules after sialidase treatment. These recycling vesicles may be responsible for the characteristically and exclusive antigen cross-presentation feature of DCs [53]. These mechanisms may have a role in the regulation of MHC-I turnover and expression at the cell surface, with the ability to insert additional MHC-I molecules on the cell membrane, which bypass de novo protein synthesis. In fact, antigenic peptide stimulation of DCs for priming of T cells has shown that cross-presented peptides are bound to recycled MHC-I molecules [54,55,56]. We can then assume that increased anti-tumoral activity and T cell-mediated cytotoxicity following sialidase treatment on DCs may be partially due to increased cross-presentation of peptides that bind to recycled, desialylated, MHC-I molecules. Thus, by blocking nascent proteins from the Golgi towards cell surface, we could observe that MHC-I show increased stability on cell membrane after desialylation, possibly being able to deliver a stronger activation signal to T cells. Furthermore, there seems to be a downregulation of *HLA-A* gene expression, possibly by negative feedback mechanisms due to increased levels at cell surface. Thus MHC-I levels at the cell surface are probably sensed by cellular mechanisms, which regulate their expression level accordingly.

It was already shown that unstimulated DCs can form immunological synapses with T cells, in the absence of antigen presentation [57]. These transient synapses can elicit changes in phosphorylation and Ca^2+^ signaling on T cells. Here, we assessed the synapse formation with T cells driven by the enzymatic removal of sialic acids from DCs surface, which was already shown to promote better cytotoxic responses. We found that desialylated DCs could establish earlier immunological synapses and activate autologous T cells, measured by the intracellular secretion of IFN-γ. We postulated that these improved immunological synapses are mediated by increased cell surface stability of MHC-I, delivering stronger signaling through the TCR for the activation of T cells. This results in a higher number of interactions and conjugates formed, which are enough to achieve the intracellular signaling level required to produce IFN-γ.

Considering these results, there seems to be evidence that the content of sialic acid on DCs’ proteins is a key regulator of their membrane stability, possibly indicating that desialylation leads to the accumulation of proteins on the cell membrane due to diminished internalization. Altogether, we infer that glycosylation has a key role in the regulation of MHC-I expression, with a shortage of sialic acids stabilizing this protein complex at the cell surface and increasing antigen presentation. Lower levels of glycosylation, and particularly sialylation of immune receptors, may lead to decreased turnover and endocytosis, increasing their expression at the cell surface.

However, other cell surface molecules bearing sialylated motifs may also pose as targets for the enzymatic removal of terminal sialic acids. Considering this, the desialylation of a regulatory surface protein on DCs may influence MHC-I expression and traffic to the cell surface, and a more detailed analysis of the mechanisms regulating MHC-I expression should be pursued.

## 5. Conclusions

The development of more efficient dendritic cell vaccines to be used as cancer immunotherapies relies on the unraveling of cellular mechanisms that can be modulated during ex vivo DCs production. Glycan engineering, particularly variation of sialic acid contents arose as a promising tool to be used in the modulation of monocyte-derived DC’s immunogenicity. Our results point to an important role of MHC-I sialic acid content in the modulation of its presence and stability at the cell surface and, hence, on the establishment of earlier and stronger synapses with T cells. Thus, modulation of sialic acid content on MHC-I may have an important contribution to the improvement of T cell function. If we understand better the underlying mechanisms and molecular targets impacted by sialic acid content, we will be closer to finding new ways to fine-tune DC’s function in clinical applications.

## Figures and Tables

**Table 1:** List of anti-MHC-I antibodies used in this study.

Antibody Clone	Specificity	Technique	Source	Reference
W6/32	Recognizes antigenic determinant common to HLA-A, B and C antigens when in their three-dimensional configuration	FC/WB and IP	ImmunoTools, Germany/Pierce, Thermo Fisher Scientific (Waltham, MA, USA)	[19]
HC10	Recognizes free HLA class I heavy chains. HC10 reacts mostly with HLA-B and HLA-C heavy chains and some HLA-A (HLA-A10, HLA-A28, HLA-A29, HLA-A30, HLA-A31, HLA-A32, HLA-A33)	FC and WB	Hybridoma supernatants	[20]
BBM.1	Recognizes both free and class I bound β2m	FC and WB	Hybridoma supernatants	[21]
BB7.2	Recognizes the α subunit of HLA-A2 which is a subset of MHC-class I molecules encoded by A*02 alleles	FC	BD Bioscience (CA, USA)	[22]
246-B8.E7	Recognizes monomorphic determinant of human MHC- I antigens (HLA-A, B, and C)	CM	Thermo Fisher Scientific (Waltham, MA, USA)	Not found

Abbreviations: MHC-I, major human histocompatibility complex class I, HLA, human leucocyte antigen, FC, Flow Cytometry; WB, Western blot; CM, Confocal Microscopy; IP, immunoprecipitation.

## Supplementary Materials

The following are available online at https://www.mdpi.com/1999-4923/12/3/249/s1, Figure S1: Ray diagram highlighting the key steps of the proteomics workflows used in this study, Figure S2: MHC-I heavy chain on human DCs is sialylated, Figure S3: Sialylated glycopeptide from HLA-A, Figure S4: Co-culture of FarRed®-labeled desialylated DCs with CFSE-labeled autologous CD8+ T cells, Table S1: List of proteins identified by nano LC-MALDI-MS/MS on SNA column elution from human DCs.
