# Supplementary material for: MHC Class I Stability is Modulated by Cell Surface Sialylation in Human Dendritic Cells
Source: Pharmaceutics. 2020 Mar 10;12(3):249. doi: 10.3390/pharmaceutics12030249 (PMC7150992; doi:10.3390/pharmaceutics12030249)
Supplement: Supplementary file 1 [file pharmaceutics-12-00249-s001.pdf]

pharmaceutics

Supplementary Materials: MHC Class I Stability is Modulated by Cell Surface Sialylation in Human Dendritic Cells

Zélia Silva [1,†], Tiago Ferro [1,2,†], Danielle Almeida [1], Helena Soares [3], José Alexandre Ferreira [4,5], Fanny M. Deschepper [1], Paul J. Hensbergen [6], Martina Pirro [6], Sandra J. van Vliet [7], Sebastian Springer [8] and Paula A. Videira [1,2,*]

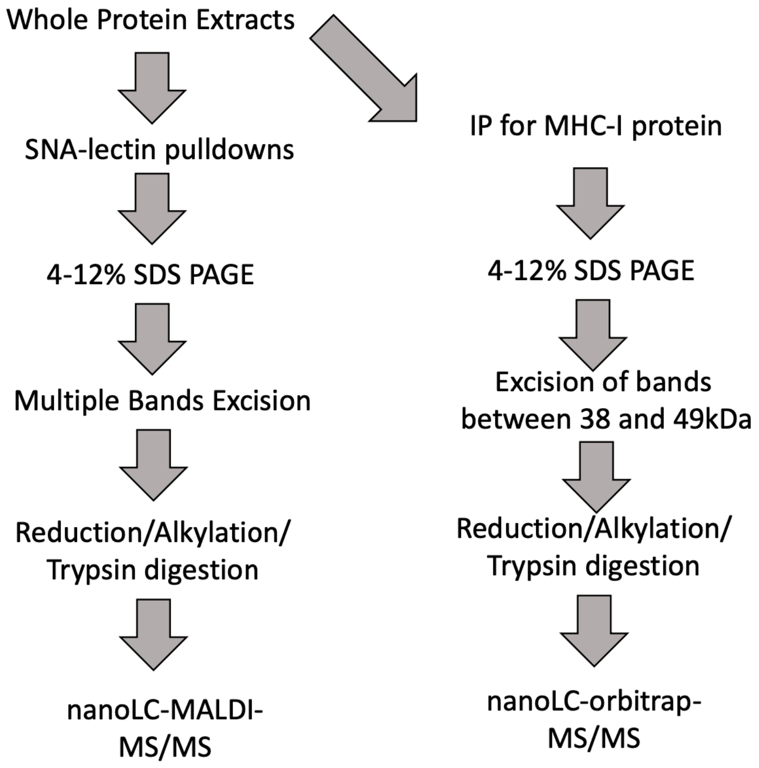

Figure S1. Ray diagram highlighting the key steps of the proteomics workflows used in this study.

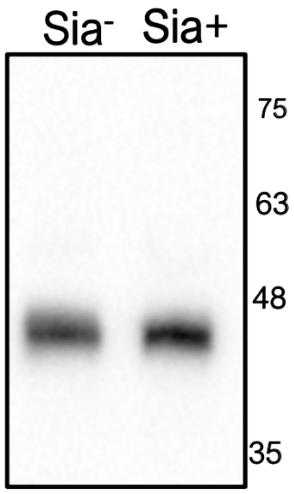

Figure S2. MHC-I heavy chain on human DCs is sialylated.

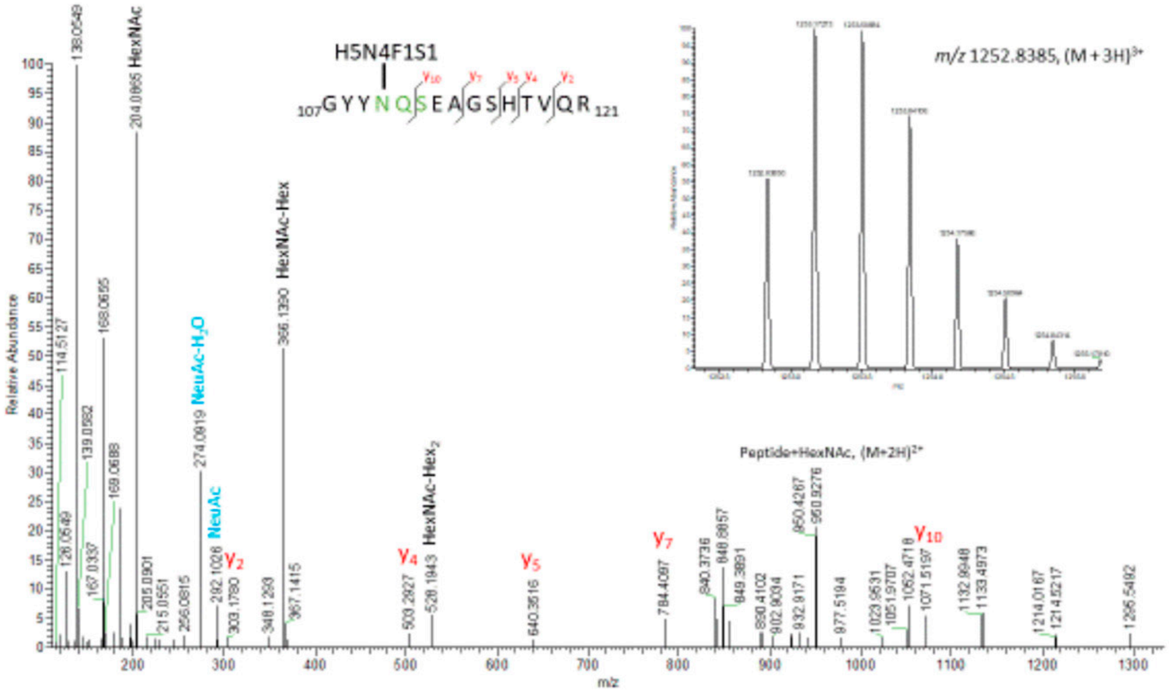

Figure S3. Sialylated glycopeptide from HLA-A.

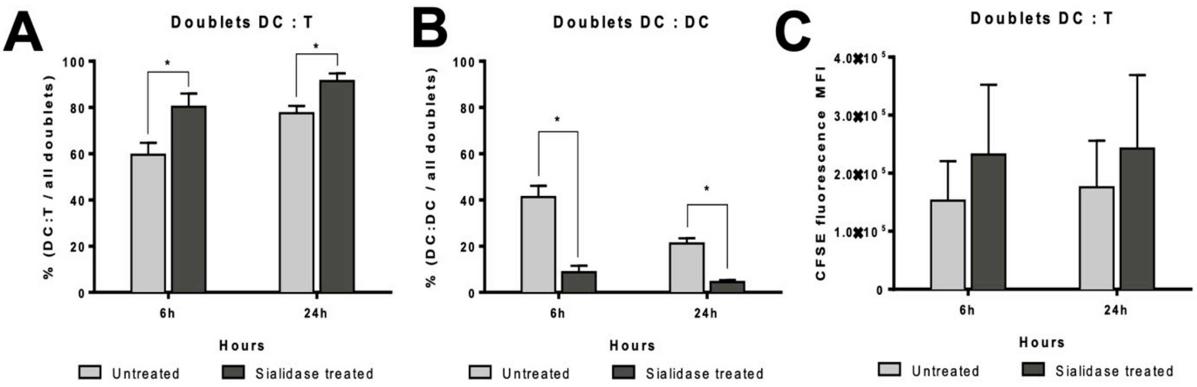

Figure S4. Co-culture of FarRed®-labeled desialylated DCs with CFSE-labeled autologous CD8+ T cells.

Table S1. List of proteins identified by nano LC-MALDI-MS/MS on SNA column elution from human DCs.

Identified Proteins	Entry name	Molecular Weight (Da)	Sequence Coverage (%)	Protein Score*
HLA class I histocompatibility antigen, A-11 alpha chain	1A11_HUMAN	40937	27	438
HLA class I histocompatibility antigen, A-23 alpha chain	1A23_HUMAN	40733	27	438
HLA class I histocompatibility antigen, B-38 alpha chain	1B38_HUMAN	40416	27	455
HLA class I histocompatibility antigen, B-59 alpha chain	1B59_HUMAN	40584	27	387
HLA class I histocompatibility antigen, A-1 alpha chain	1A01_HUMAN	40846	26	380
HLA class I histocompatibility antigen, A-36 alpha chain	1A36_HUMAN	40934	26	380
HLA class I histocompatibility antigen, B-7 alpha chain	1B07_HUMAN	40460	25	443
HLA class I histocompatibility antigen, B-42 alpha chain	1B42_HUMAN	40333	25	443
HLA class I histocompatibility antigen, B-39 alpha chain	1B39_HUMAN	40328	24	396
HLA class I histocompatibility antigen, B-67 alpha chain	1B67_HUMAN	40342	24	396
HLA class I histocompatibility antigen, B-55 alpha chain	1B55_HUMAN	40496	23	328
HLA class I histocompatibility antigen, B-56 alpha chain	1B56_HUMAN	40478	23	328
HLA class I histocompatibility antigen, B-82 alpha chain	1B82_HUMAN	40421	23	328
HLA class I histocompatibility antigen, B-13 alpha chain	1B13_HUMAN	40474	22	366
HLA class I histocompatibility antigen, B-44 alpha chain	1B44_HUMAN	40481	22	366
HLA class I histocompatibility antigen, B-53 alpha chain	1B53_HUMAN	40495	22	366
HLA class I histocompatibility antigen, A-25 alpha chain	1A25_HUMAN	41218	21	328
HLA class I histocompatibility antigen, A-25 alpha chain	1A26_HUMAN	41218	21	328
HLA class I histocompatibility antigen, A-32 alpha chain	1A32_HUMAN	41048	21	298
HLA class I histocompatibility antigen, A-34 alpha chain	1A34_HUMAN	41055	21	328
HLA class I histocompatibility antigen, A-66 alpha chain	1A66_HUMAN	41082	21	238
HLA class I histocompatibility antigen, A-74 alpha chain	1A74_HUMAN	40891	21	292
HLA class I histocompatibility antigen, B-14 alpha chain	1B14_HUMAN	40358	21	337
HLA class I histocompatibility antigen, B-8 alpha chain	1B08_HUMAN	40331	20	361
HLA class I histocompatibility antigen, B-27 alpha chain	1B27_HUMAN	40428	20	314
HLA class I histocompatibility antigen, B-40 alpha chain	1B40_HUMAN	40505	20	400
HLA class I histocompatibility antigen, Cw-12 alpha chain	1C12_HUMAN	40886	20	397
HLA class I histocompatibility antigen, Cw-15 alpha chain	1C15_HUMAN	40863	20	397
HLA class I histocompatibility antigen, A-29 alpha chain	1A29_HUMAN	40863	19	203
HLA class I histocompatibility antigen, B-15 alpha chain	1B15_HUMAN	40388	19	307
HLA class I histocompatibility antigen, B-35 alpha chain	1B35_HUMAN	40455	19	307
HLA class I histocompatibility antigen, B-46 alpha chain	1B46_HUMAN	40440	19	307
HLA class I histocompatibility antigen, B-47 alpha chain	1B47_HUMAN	40571	19	307
HLA class I histocompatibility antigen, B-49 alpha chain	1B49_HUMAN	40581	19	353
HLA class I histocompatibility antigen, B-51 alpha chain	1B51_HUMAN	40566	19	307
HLA class I histocompatibility antigen, B-52 alpha chain	1B52_HUMAN	40521	19	307

HLA class I histocompatibility antigen, A-80 alpha chain	1A80_HUMAN	40792	18	209
HLA class I histocompatibility antigen, B-73 alpha chain	1B73_HUMAN	40435	18	296
HLA class I histocompatibility antigen, A-43 alpha chain	1A43_HUMAN	41033	17	278
HLA class I histocompatibility antigen, A-68 alpha chain	1A68_HUMAN	40909	17	310
HLA class I histocompatibility antigen, A-69 alpha chain	1A69_HUMAN	40977	17	310
HLA class I histocompatibility antigen, B-41 alpha chain	1B41_HUMAN	40539	17	341
HLA class I histocompatibility antigen, B-48 alpha chain	1B48_HUMAN	40362	17	238
HLA class I histocompatibility antigen, B-81 alpha chain	1B81_HUMAN	40400	17	238
HLA class I histocompatibility antigen, Cw-6 alpha chain	1C06_HUMAN	40969	17	384
HLA class I histocompatibility antigen, Cw-8 alpha chain	1C08_HUMAN	40773	17	337
T-cell surface glycoprotein CD1b	CD1B_HUMAN	36939	17	268
Putative HLA class I histocompatibility antigen, alpha chain H	HLAH_HUMAN	40892	17	280
HLA class I histocompatibility antigen, B-37 alpha chain	1B37_HUMAN	40456	16	293
HLA class I histocompatibility antigen, B-45 alpha chain	1B45_HUMAN	40414	16	293
HLA class I histocompatibility antigen, B-50 alpha chain	1B50_HUMAN	40541	16	293
HLA class I histocompatibility antigen, B-78 alpha chain	1B78_HUMAN	40478	16	248
HLA class I histocompatibility antigen, Cw-16 alpha chain	1C16_HUMAN	40753	16	290
HLA class I histocompatibility antigen, B-54 alpha chain	1B54_HUMAN	40380	15	258
HLA class I histocompatibility antigen, B-57 alpha chain	1B57_HUMAN	40224	15	258
HLA class I histocompatibility antigen, B-58 alpha chain	1B58_HUMAN	40337	15	258
HLA class I histocompatibility antigen, A-33 alpha chain	1A33_HUMAN	40892	14	186
HLA class I histocompatibility antigen, Cw-2 alpha chain	1C02_HUMAN	41095	14	325
HLA class I histocompatibility antigen, A-2 alpha chain	1A02_HUMAN	40922	13	202
HLA class I histocompatibility antigen, A-3 alpha chain	1A03_HUMAN	40841	13	202
HLA class I histocompatibility antigen, A-24 alpha chain	1A24_HUMAN	40689	13	277
HLA class I histocompatibility antigen, A-30 alpha chain	1A30_HUMAN	40905	13	277
HLA class I histocompatibility antigen, A-31 alpha chain	1A31_HUMAN	41004	13	196
HLA class I histocompatibility antigen, B-18 alpha chain	1B18_HUMAN	40275	13	234
HLA class I histocompatibility antigen, Cw-5 alpha chain	1C05_HUMAN	40912	13	231
HLA class I histocompatibility antigen, Cw-7 alpha chain	1C07_HUMAN	40649	13	285
HLA class I histocompatibility antigen, Cw-3 alpha chain	1C03_HUMAN	40861	10	218
HLA class I histocompatibility antigen, Cw-4 alpha chain	1C04_HUMAN	40995	10	265
HLA class I histocompatibility antigen, Cw-14 alpha chain	1C14_HUMAN	40838	10	265
HLA class I histocompatibility antigen, Cw-18 alpha chain	1C18_HUMAN	40933	10	265
HLA class I histocompatibility antigen, Cw-17 alpha chain	1C17_HUMAN	41238	9	132
HLA class I histocompatibility antigen, Cw-1 alpha chain	1C01_HUMAN	40965	7	130
T-cell surface glycoprotein CD1a	CD1A_HUMAN	37077	7	148
HLA class II histocompatibility antigen, DRB1-1 beta chain	2B11_HUMAN	29914	5	35
HLA class II histocompatibility antigen, DRB1-4 beta chain	2B14_HUMAN	30112	5	35
HLA class II histocompatibility antigen, DRB1-7 beta chain	2B17_HUMAN	29822	5	35
HLA class II histocompatibility antigen, DRB1-8 beta chain	2B18_HUMAN	30004	5	35

HLA class II histocompatibility antigen, DRB1-9 beta chain	2B19_HUMAN	29826	5	35
HLA class II histocompatibility antigen, DRB1-10 beta chain	2B1A_HUMAN	30002	5	35
HLA class II histocompatibility antigen, DRB1-11 beta chain	2B1B_HUMAN	30160	5	35
HLA class II histocompatibility antigen, DRB1-16 beta chain	2B1G_HUMAN	30030	5	35
T-cell surface glycoprotein CD1c	CD1C_HUMAN	37654	5	115
HLA class II histocompatibility antigen, DR beta 3 chain	DRB3_HUMAN	29962	5	35
HLA class II histocompatibility antigen, DR beta 5 chain	DRB5_HUMAN	30056	5	35
Ig gamma-1 chain C region	IGHG1_HUMAN	36106	5	44
Ig gamma-2 chain C region	IGHG2_HUMAN	36901	5	820
Lymphocyte-specific protein 1	LSP1_HUMAN	37192	5	72
HLA class I histocompatibility antigen, alpha chain E	HLAE_HUMAN	40157	4	99
HLA class I histocompatibility antigen, alpha chain G	HLAG_HUMAN	38224	4	99
Ig gamma-3 chain C region	IGHG3_HUMAN	41287	4	82
HLA class I histocompatibility antigen, alpha chain F	HLAF_HUMAN	39062	3	59
CD44 antigen	CD44_HUMAN	81538	2	67
Integrin beta-2	ITB2_HUMAN	84782	2	55
Integrin alpha-M	ITAM_HUMAN	127179	1	67

* Significant protein scores ($p<0.05$).
